# Gout and ‘Podagra’ in medieval Cambridge, England

**DOI:** 10.1016/j.ijpp.2021.04.007

**Published:** 2021-06

**Authors:** Jenna M. Dittmar, Piers D. Mitchell, Peter M. Jones, Bram Mulder, Sarah A. Inskip, Craig Cessford, John E. Robb

**Affiliations:** a McDonald Institute for Archaeological Research, University of Cambridge, Cambridge, UK; b Department of Archaeology, University of Aberdeen, Aberdeen, UK; c Department of Archaeology, University of Cambridge, Cambridge, UK; d King’s College, University of Cambridge, Cambridge, UK; e School of Archaeology and Ancient History, University of Leicester, Leicester, UK; f Cambridge Archaeological Unit, Department of Archaeology, University of Cambridge, Cambridge, UK

**Keywords:** Crystal arthropathy, Inflammatory arthritis, Micro-computed tomography (μCT), Hallux valgus, Diet, Social status

## Abstract

**Objective:**

To estimate the prevalence rate of gout and to explore the social factors that contributed to its development in the various sub-populations in medieval Cambridge.

**Materials:**

177 adult individuals from four medieval cemeteries located in and around Cambridge, UK.

**Methods:**

Lesions were assessed macroscopically and radiographically. Elements with lytic lesions were described and imaged using micro-computed tomography (μCT) to determine their morphology.

**Results:**

Gout was identified in 3 % of the population. Individuals buried in the friary had highest prevalence (14 %), with low prevalence rates in the Hospital (3 %) and town parish cemetery (2 %), with no cases in the rural parish cemetery. Gout was more prevalent during the 14th–15th centuries than the 10th–13th centuries.

**Conclusion:**

The high prevalence rate of gout in the friary is at least partly explained by the consumption of alcohol and purine-rich diets by the friars and the wealthy townsfolk. Medieval medical texts from Cambridge show that gout (known as podagra) was sometimes treated with medications made from the root of the autumn crocus. This root contains colchicine, which is a medicine that is still used to treat gout today.

**Significance:**

This is one of the first studies to assess the epidemiology of gout in medieval England and suggests that gout varied with social status.

**Limitations:**

Our sample size precludes statistical analysis.

**Suggestions for further research:**

Additional studies that assess the epidemiology of gout in medieval Europe is needed in order to be able to fully contextualize these findings.

## 1 Introduction

Gout is an inflammatory arthritis resulting from monosodium urate crystals forming in soft tissues or joints. These trigger an inflammatory response, classically in the first metatarsophalangeal joint (MTPJ) of the feet (Joosten et al., 2010; Neogi et al., 2015). There are four clinical phases of gout: asymptomatic hyperuricemia, acute gouty arthritis, intercritical gout, and chronic tophaceous gout (Gentili, 2003; Kobayshi et al., 2005). Gout attacks are characterized by the sudden onset of burning joint pain, with associated swelling that is tender to touch that can persist for up to three weeks (Roddy, 2011). The intense pain during an attack can cause physical impairment that has a major impact on health-related quality of life (Kim et al., 2014; Rome et al., 2011). Attacks may be triggered by a large meal of purine-rich foods, alcohol consumption (especially beer), or fasting (Joosten et al., 2010). The severity and frequency of these attacks can increase over time until an individual develops chronic tophaceous gout, which is characterized by chronic pain and stiffness, joint damage and erosive arthropathy (Roddy, 2011). While gouty arthritis most commonly affects the metatarsophalangeal joint of the great toe, it can affect any joint including those in the axial skeleton (Gentili, 2006; Roddy, 2011). This condition is associated with reduced quality of life, functional impairment, reduced productivity and a higher risk of death (Singh and Strand, 2008).

Although relatively limited research has been conducted on gout, the consumption of alcohol and protein-rich diets has been cited as a reason to explain the high prevalence rate in certain subsets of past societies. Cases of gout have been identified in those that lived alternative lifestyles (such as living in certain religious institutions) (Gilchrist and Sloane, 2005; Randerson et al., 2015; Swinson et al., 2010) and those of high status such as members of the Medici family, the Grand Dukes of Renaissance Florence (Fornaciari et al., 2009, 2019). Underlying genetic composition has also been shown to play a substantial role in the development of gout (Kuo et al., 2015). Research that investigated the epidemiology of gout in populations from the Pacific islands, where this disease is particularly common today (Buckley, 2011), found a prevalence of 6 % in 950–1450 AD Guam (Rothschild and Heathcote, 1995) and 35 % in 2,000 BC Vanuatu (Buckley, 2007).

The limited existing work on gout in medieval Britain has suggested its prevalence is around 1% (Rogers, 2000; Waldron, 2007). However, much of the research on this condition has been the presentation of isolated cases, and systematic research to explore the prevalence rate of gout across the population of a medieval town has never before been conducted. As such, it is unclear what role social and environmental factors had in the expression of gout on a population-level. The aim of this paper is to compare the prevalence rates of gout in various subpopulations from medieval Cambridge. Within this research, we examine individuals who were buried in a parish cemetery (which was the normative burial site for the majority of the population), a friary, a charitable institution for the poor, and a rural parish located approximately 6 km from the town.

### 1.1 Medieval Cambridge

In the High/Late medieval period, Cambridge was a medium-sized market town with a population of about 3500 in 1279−80 (Casson et al., 2020: 318). Farming and river trade up and down the Cam formed the basis of economic life. The import and export of goods was enabled by access to the North Sea via the River Cam and the Great Ouse. Medieval Cambridge was also home to numerous ecclesiastical institutions, including the university. Although founded in *c.* 1208–1210, the university was not a major component of the town until the late 13th to early 14th centuries. Before this, it comprised relatively small individual elements within the larger town and surrounding fields (Cessford, forthcoming a). A significant clerical population that was distinct from the university was also present within the town. The members of various religious orders were based in a range of friaries, the Hospital of St John the Evangelist, the Benedictine nunnery of St Radegund, plus the outlying Barnwell priory and nearby leprosarium (Cessford, forthcoming a).

Due to the influx of people drawn to Cambridge, the town had a unique and varied social landscape that included townspeople as well as members of religious orders and the university. Townspeople primarily consisted of laborers including general laborers, agricultural workers and tradesmen with some pursuing specialized trades (see Dyer, 2009; Miller and Hatcher, 2014). This category also includes a small number of prosperous families with extensive properties and servants as well as the urban poor and needy, which may have included both chronically impoverished people and people driven into poverty by loss of livelihood and family support networks. Unlike most other medieval towns, a major adult subgroup would have been members of religious foundations such as friaries and colleges, who experienced a specialized lifestyle and diet governed by specified institutional rules (Ellis and Salzman, 1948).

## 2 Materials & methods

### 2.1 Materials

**Fig. 1:**
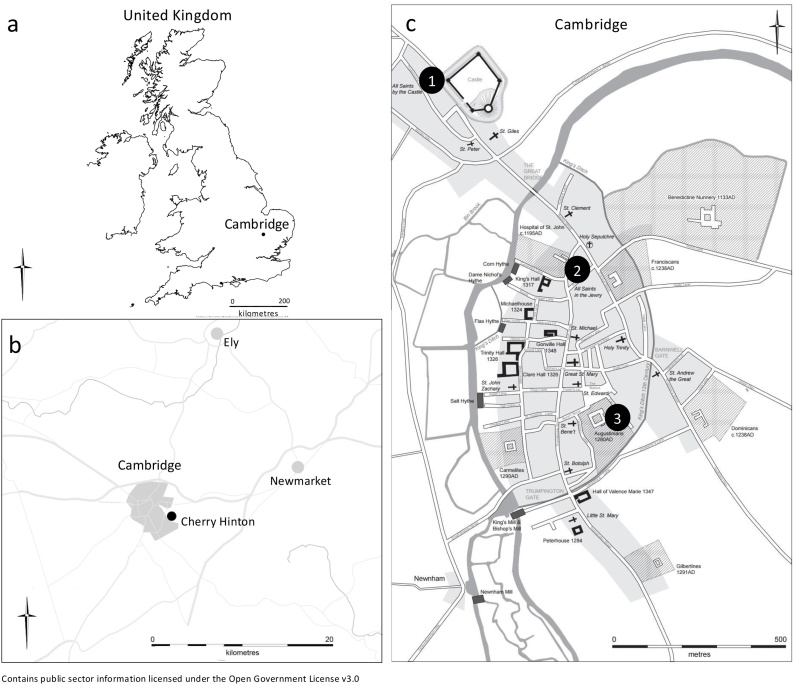
a) Map of the United Kingdom showing the location of Cambridge, b) Map of the area surrounding Cambridge showing the location of Cherry Hinton, c) Map of Cambridge c. 1350 showing the location of 1) All Saints by the Castle parish burial ground, 2) the detached burial ground of Hospital of St John the Evangelist and 3) the burial ground of the Augustinian friary (map created by Vicki Herring).

As part of the ‘After the Plague: Health and History in Medieval Cambridge’ project, human skeletal remains of adult individuals (n = 177) from the High/Late medieval period were analyzed. These individuals came from four sites located in and around Cambridge that each represent a different social context (Fig. 1). The skeletal remains from the parish cemetery of All Saints by the Castle (n = 50), represent the majority of the townsfolk who were buried in such cemeteries. This parish was socio-economically mixed but generally rather poorer than the town as a whole (Casson et al., 2020: 143-4). Those buried at the Hospital of St John the Evangelist (n = 69) predominantly represents inmates of a charitable hospital, while those from the Augustinian friary (n = 21) represent members of the clergy and the relatively wealthy laity. The burial ground associated with the proprietary church at Church End in Cherry Hinton (n = 37), represents the rural poor. Gout primarily affects older adults: the onset before age 40 years is highly unusual (Kuo et al., 2015). Therefore, this study was limited to adult individuals (> 18 years of age at the time of death) that had at least one first metatarsal present.

Each skeleton was dated based on the overall periods when the burial grounds are documented as being in use, plus the skeletons’ stratigraphic location with the burial sequences at the sites. Those from the Hospital of St John the Evangelist were also radiocarbon dated. In addition to standard calibration, allowance has been made for various factors that can affect radiocarbon determinations on human bone making them appear older than the date an individual died and that are not covered by standard calibration, such as marine dietary offset, bone turnover during life (for a full methodological description see Cessford and Alexander, forthcoming).

#### 2.1.1 The parish church of All Saints by the Castle

All Saints by the Castle church is thought to have been founded c. 940–1100, and the cemetery closed when the parish was amalgamated with nearby St Giles in 1365/6. Four areas of the graveyard were excavated in 1973 (Craddock and Gregory, 1975), with further skeletons uncovered in 1988 and 1994 (Cessford and Dickens, 2005a). In total around 215 skeletons have been excavated, in various states of completeness. It can be described as ‘rurban’, being a mixed rural and urban parish. The parish population comprised a cross-section of social classes, which was probably broadly representative of Cambridge as a whole.

#### 2.1.2 The Hospital of St John the Evangelist

The Hospital of St John the Evangelist was established *c.* 1190–1200 to provide care for the poor and infirm of Cambridge. The Hospital remained in use until 1511 when it was dissolved to create St John’s College. The detached cemetery, that was established by 1204–14, was excavated by the Cambridge Archaeological Unit during 2010–11. About 400 complete and partial in situ burials were excavated (Cessford, 2015). Both men and women were buried in the cemetery, but there were no children under the age of five (Cessford, 2015: 101). Those buried there were predominantly hospital inmates, who were poor and unable to be cared for by family members. The burial ground also contained a small number of ‘corrodians’ who were wealthy elderly lay people who paid to live in a religious institution for the rest of their lives, as well as a few local benefactors who made small to moderate donations to the Hospital (Cessford, forthcoming b).

#### 2.1.3 The Augustinian friary

This friary was established around 1279/80–89 and remained in use until the Dissolution of the Monasteries in 1538. Parts of the friary were excavated in 1908–09, followed by more extensive excavations in 2016–19 by the Cambridge Archaeological Unit. Excavated burials include 32 skeletons in a cemetery to the south of the friary church dating to *c.* 1290–mid/late 14th century, and six in a chapter house dating to mid/late 14th century–1538 (Cessford, 2017). These individuals represent a mixture of members of the Augustinian order and lay benefactors. These burials were differentiated archaeologically as friars appear to have been buried clothed and wearing a belt with buckle; whilst lay people were typically buried wrapped in a shroud (Cessford, 2017). The townspeople buried in the friary likely represent some of the more prosperous members of Cambridge society, as typically a donation was required for burial within the friary. Eleven friars and ten lay people from the friary cemetery were included in this study.

#### 2.1.4 The proprietary church at Church End, Cherry Hinton

The church and cemetery at Church End, Cherry Hinton, was located about 6 km southeast of Cambridge. It was excavated by the Hertfordshire Archaeological Trust in 1999 (Cessford and Dickens, 2005b; Lally, 2008; McDonald and Doel, 2000). Over 670 graves were excavated and the remains of around 980 individuals (including the disarticulated material) were identified. The burials at Cherry Hinton were west-east aligned extended supine inhumations, in simple earth cut graves with the head to the west and most individuals were probably buried in shrouds. Apart from four adults with accompanying neonates there were no identifiable multiple burials. Disarticulated human remains were present in some grave fills, but these were not included in the study. The cemetery was associated with a rural estate starting around 940–1000 and continuing in use until *c*. 1120–70. Those buried are believed to represent a cross-section of the various types of free and unfree peasants of this predominantly agricultural site. As part of the ‘After the Plague’ project, a subset of individuals from this site (n = 124) were analyzed. Of these, 37 adults with surviving feet were included in this study.

### 2.2 Methods

Each skeleton was assigned a ‘project specific number’ (PSN) that served as a unique identifier for the ‘After the Plague’ project. Individuals are referred to by these numbers throughout. The human skeletal remains in this study were fully assessed following internationally accepted guidelines (Buikstra and Ubelaker, 1994; Mitchell and Brickley, 2017). As part of this analysis, each joint surface was carefully examined, including those in the feet and hands. If lytic lesions were identified on the joint surfaces, a differential diagnosis was conducted (see Sections 2.2.1 and 2.2.2). The biological sex of each adult skeleton was estimated by examining the sexually dimorphic characteristics of the pelvis and cranium (Buikstra and Ubelaker, 1994; Phenice, 1969; Schwartz, 2007). Genetic sex was determined using aDNA analysis (see Inskip et al., 2019). Age-at-death was estimated using the degenerative changes observable on the pubic symphysis (Brooks and Suchey, 1990); auricular surface (Buckberry and Chamberlain, 2002) and sternal rib ends (İşcan et al., 1984, 1985) and the sternal end of the clavicle (Falys and Prangle, 2015). Individuals were divided into the following age categories: young adult (18–25 years), middle adult (26–44 years), mature adult (45–59 years), and old adult (60+ years). If age-at-death could not be determined due to incompleteness or damage, individuals with complete epiphyseal fusion were classified as ‘adult’. See Table 1 for demographic data for the individuals in this study.

**Table 1:** Frequency table for biological sex and age-at-death categories by site.

Age	Male	Female	Unobservable, sex unknown	Total
**All Saints by the Castle**				
Young adult	2	1	0	3
Middle adult	6	4	0	10
Mature adult	7	5	0	12
Old adult	5	5	1	11
Adult	1	0	13	14
**Total**	**21**	**15**	**14**	**50**

**Hospital of St John the Evangelist**				
Young adult	3	5	0	8
Middle adult	14	5	0	19
Mature adult	10	5	0	15
Old adult	3	1	0	4
Adult	1	2	20	23
**Total**	**31**	**18**	**20**	**69**

**Augustinian friary**				
Young adult	2	0	0	2
Middle adult	7	0	0	7
Mature adult	4	0	0	4
Old adult	2	1	0	3
Adult	1	0	4	5
**Total**	**16**	**1**	**4**	**21**

**Church End, Cherry Hinton**				
Young adult	0	0	0	0
Middle adult	8	7	0	15
Mature adult	8	3	0	11
Old adult	1	1	0	2
Adult	3	1	5	9
**Total**	**20**	**12**	**5**	**37**

#### 2.2.1 Criteria used to identify gout in skeletal remains

In gout, sodium urate crystals are deposited in connective tissues adjacent to joints and in bone marrow. The deposition of these crystals elicits an inflammatory response, which leads to erosion of bone. By far the most commonly affected joint in the body is the metatarsophalangeal joint (MTPJ) of the great toe, especially its medial side. In the diagnostic criteria for gout created by the American College of Rheumatology and European League Against Rheumatism (Neogi et al., 2015) the presence of tophi is specific to gout (when compared to rheumatoid arthritis, seronegative arthropathies, septic arthritis and pseudo-gout) and seems to be the most useful criterion to identify gout. These sodium urate tophi usually dissolve as part of the decomposition process after death, so are generally not present in excavated human skeletal remains unless unusually favorable taphonomic conditions exist. Occasionally uric acid crystals may be preserved as a white powder adherent to the bone, which has been shown to be uric acid when examined using microscopy for birefringent crystals or with high performance liquid chromatography (Swinson et al., 2010). This type of analysis was not undertaken as part of this study.

The characteristic skeletal changes of chronic tophaceous gout in archaeological remains are well-defined lytic lesions which are ‘scooped-out’ in appearance, often with a thin overhanging hook-like sclerotic margin (Ortner, 2003; Rogers et al., 1987; Rothschild and Heathcote, 1995). The overhanging edge is caused by the gradual expansion of the tophus which has eroded the cortex of the bone. The base of the cavity in chronic gout will generally have a degree of cortication. While typically located adjacent to the joint, the lesion may affect the articular surface and extend onto the shaft of the bone. The morphology of the lesions and the presence of reactive bone formation surrounding the lesion, coupled with the skeletal distribution across the skeleton, generally distinguishes the erosive lesions caused by gout from those from other arthropathies (see Section 2.2.2).

Skeletal elements with pathological lesions that were suggestive of gout were recorded and analyzed further using the Nikon XT H 225 ST microCT scanner at the Cambridge Biotomography Centre. The skeletal elements were placed in an acrylic container and scanned at a resolution of 125 μm. The energy source settings were kept constant at 140 kV and 100 μA, with an exposure of 708 ms. For each of the 1080 projections 2 frames were taken and averaged. Cross-sectional slices were reconstructed from the scans in CT-Pro software (Nikon Metrology) and exported as TIFF-stack. Individual bones were isolated from the stack in Avizo 9.0.1 (Thermo Fisher Scientific), and reoriented anatomically based on their 3D isosurface, to allow analysis of the lytic lesions in a suitable plane. The skeletal lesions observed were compared to CT images of the bony lesions of gout in the clinical literature (Gentili, 2003, 2006; Dalbeth et al., 2009; Girish et al., 2013).

#### 2.2.2 Differential diagnosis of other conditions affecting the 1st metatarsophalangeal joint

The two main groups of conditions that can mimic the lesions of gout in human remains are hallux valgus and inflammatory arthropathies. Hallux valgus is lateral angulation of the great toe commonly casued by wearing tight fitting shoes with a pointed toe box. Inflammation at the insertion of the MTPJ collateral ligaments and sesamoid ligaments can trigger lytic cavitating lesions on the medial side of the metatarsal head (Mays, 2005; Dittmar et al., 2021), which can mimic early gout. If other characteristics of hallux valgus are present, such as a laterally angulated MTPJ joint surface, a ridge on the medial side of the joint surface, degenerative change at the sesamoid bones, then hallux valgus is the most likely diagnosis. However, when none of these further signs are present, cavitation on the medial side of the metatarsal head needs to be carefully assessed to see if it is located at the attachments of these ligaments (suggesting hallux valgus) or in other locations (suggesting gout). Diagnosis is complicated by the fact that these conditions are not mutually exclusive and hallux valgus and gout commonly co-occur.

Inflammatory arthropathies may be seropositive (rheumatoid) or seronegative (such as psoriatic arthritis, ankylosing spondylitis, reactive arthritis, enteropathic arthritis, and erosive osteoarthritis) (Rogers and Waldron, 1995). Lesions of inflammatory arthropathy are located on the margins of articular surfaces, erode into the joint surface, affect both proximal and distal surfaces of the joint, and may be associated with other lesions such as erosion (rheumatoid) or fusion (ankylosing spondylitis, psoriatic arthritis) of the spine. Several of these conditions commonly involve the hands and feet including the interphalangeal joints, but do not involve the great toe with higher prevalence than other digits (Zias and Mitchell, 1996; Samsel et al., 2014). Septic arthritis of the first MTPJ of the toe may lead to joint destruction, osteomyelitis in adjacent bones, or fusion after healing. Classical osteoarthritis can also lead to degenerative change in the joint (hallux rigidus) or joint fusion. Table 2 indicates the conditions that were considered in the differential diagnosis (see Table 3).

**Table 2:** Descriptions of conditions that should be included in the differential diagnosis for gout.

Condition	Description of skeletal involvement	References
Gout	Asymmetric erosive arthritis that primarily affects the peripheral joints. Although rare, skeletal changes can also be present in the axial skeleton. Lesions are well-defined and are ‘scooped-out’ in appearance, often with a thin, overhanging, hook-like sclerotic margins. The first metatarsophalangeal joint is commonly affected but any joint can be affected.	Ortner (2003), Rogers et al. (1987), Rothschild and Heathcote (1995)
Rheumatoid arthritis	An autoimmune disorder of unknown aetiology that causes symmetrical erosive polyarthritis that most often affects the synovial joints of the appendicular skeleton including the small joints of the hands and feet. Most commonly the metacarpophalangeal joints and the metatarsophalangeal joints and the proximal interphalangeal joints are affected, but the distal interphalangeal joints are rarely affected. Ankylosis of joints in the hands and feet can occur in severe cases.	Rogers et al. (1987), Ortner (2003), Waldron (2019)
Erosive osteoarthritis	Asymmetrical arthropathy that produces both proliferative and erosive changes along with ankylosis. Metacarpals as well as proximal and distal interphalangeal joints of the hands are most commonly affected. Other joints are rarely affected.	Rogers et al. (1987), Waldron (2019)
Psoriatic arthritis	An asymmetric erosive arthritis associated with psoriasis, which is usually negative for rheumatoid factor. Commonly affects four joints or fewer. The involvement of the spinal joints and sacroiliac joints is typical and enthesopathy is common. Erosive lesions in the distal interphalangeal joints of the hands are common and these lesions are often accompanied by proliferative lesions located at erosion margins. The “pencil-in-cup” deformity (when the tip of a bone becomes pointed like a pencil) of the distal interphalangeal joints is characteristic.	Rogers et al. (1987), Rogers (2000), Sudoł-Szopińska et al. (2016), Waldron (2019)
Reactive arthritis	A form of reactive arthritis that occurs in reaction to an infection by certain bacteria elsewhere in the body. Sacroiliac involvement (bilateral or unilateral) is common and paravertebral bridges may cause spinal fusion with skip lesions. Asymmetrical erosive changes to the small joints can occur in the feet and less commonly, the hands. Erosion of the metatarsophalangeal joints and of the metatarsal heads is relatively common. Lanois deformity can occur in the feet.	Waldron (2019), Martel et al. (1979)
Hallux valgus	Hallux valgus is lateral angulation of the great toe. Inflammation at the insertion of the metatarsophalangeal collateral ligaments and sesamoid ligaments can trigger lytic cavitating lesions on the medial side of the metatarsal head. Characteristics of hallux valgus include: laterally angulated first metatarsophalangeal joint surface, a ridge on the medial side of the distal articular surface, degenerative changes to the distal articular surface and/or sesamoid bones. Lesions on the medial aspect of the head tend to have rounded margins and do not affect the articular surface of the first metatarsophalangeal joint.	Mays (2005)

**Table 3:** Description of lesions observed on individuals from Cambridge and differential diagnosis.

PSN	Age and Sex	Affected elements	Description of Lesions	Differential diagnosis	Final Diagnosis
42	Middle adult male	Left first metatarsal	Well-defined, cavernous lytic lesion with thin, overhanging sclerotic margins present on the medial aspect of the head, adjacent to the joint surface (Fig. 4a, c). The distal joint surface is laterally deviated with an anterolateral extension of the joint surface (Fig. 4b, f, indicated by grey arrow). There is also a ridge on the distal articular surface of the head, located approximately 3 mm from the medial margin of the articular surface (Fig. 4f, indicated by white arrow).	The observed lesions are symmetrical, but only the medial aspects of the heads of the first metatarsals are affected. This distribution is inconsistent with rheumatoid arthritis. There are no proliferative changes or ankylosis present so unlikely to be erosive osteoarthritis. There is no involvement of the sacroiliac joints or spine, therefore it is unlikely to be psoriatic arthritis and is less likely to be reactive arthritis. The ridges on the distal articular surface of each metatarsal are consistent with lateral deviation of the first proximal pedal phalanx.	Both gout and hallux valgus
		Right first metatarsal	Well-defined, cavernous lytic lesion with thin, overhanging sclerotic margins present on the medial aspect of the head, adjacent to the joint surface (Fig. 4e). The distal joint surface is laterally deviated (Fig. 4d and there is a ridge located on the distal articular surface of the head, located approximately 2−3 mm from the medial margin of the articular surface.		
93	Mature adult male	Left first metatarsal	Large, scooped-out lytic lesion with thin overhanging sclerotic margins located on the medial aspect of the head. The lesion is primarily located adjacent to the joint surface, but some of erosion of the medial aspects of the articular surface with signs of overlying taphonomic damage is present (Fig. 3b). New reactive bone formation surrounds the lesion.	The distribution of the lesions is symmetrical, but lesions are only present in the feet. This is uncommon with rheumatoid arthritis and erosive osteoarthritis, which more commonly affect the hands. The sacroiliac joints are not affected, but vertebral fusion is present (see below). Psoriatic arthritis and reactive arthritis are considered below.	Gout
		Right first metatarsal	Scooped-out lytic lesions with sclerotic margins that have coalesced located on the medial aspect of the head with evidence of erosion of the medial and dorsal aspects of the articular surface.		
		Right first proximal pedal phalanx	Multiple scooped-out lytic lesions with sclerotic margins that have coalesced present on the medial aspect of the head with evidence of erosion of the medial and planter aspects of the articular surface (Fig. 3b).		
		Right first distal pedal phalanx	Well-defined scooped-out lytic lesion with thin overhanging margins located on the dorsal margin of the proximal articular surface.		
		Thoracic vertebrae	T6-9 ankylosed with extensive bone proliferation located on the right side of the vertebral column. These are ‘dripping candle wax’ in appearance. T10 also has adhering bone proliferation present on the right aspect of the vertebral body but remains unfused. The vertebral disc space is preserved. No sacroiliac involvement.	The preserved joint spaces and ‘dripping candle wax’ ossifications that are found exclusively on the right side of the affected vertebra is diagnostic of DISH. There is no evidence of sacroiliac joint fusion and the morphology of the changes is inconsistent with ankylosing spondylitis, reactive arthritis and psoriatic arthritis.	DISH
522	Old adult male	Right first metatarsal	Multiple scooped-out lytic lesions with sclerotic margins that have coalesced resulting in significant erosion to the medial aspect of the head and the distal articular joint surface (Fig. 2).	The distribution of lesions is symmetrical but confined to the pedal elements. There is no evidence of erosive lesions in the joints of the hands or elsewhere in the skeleton. This distribution of lesions observed here is uncommon in rheumatoid arthritis as the distal phalanges tend to not be affected. There are no proliferative changes, so it is unlikely to be erosive osteoarthritis. There is no involvement of the sacroiliac joints or spine, so it is unlikely to be psoriatic arthritis or reactive arthritis. The distribution and erosive nature of the lesions is inconsistent with hallux valgus.	Gout
		Left first metatarsal	Multiple scooped-out lytic lesions with sclerotic margins that have coalesced located on the medial aspect of the head. The distal articular surface has been affected on the medial and planter aspects (Fig. 3a).		
		Right first proximal pedal phalanx	Well-defined, erosive lesions with sclerotic margins on the dorsal surface of the element, immediately adjacent to the proximal articular surface.		
		Right first distal pedal phalanx	Small (2 mm in diameter), scooped-out lytic lesion with overhanging, sclerotic margins located on the dorsal aspect immediately adjacent to the proximal articular surface.		
		Right 5^th^ metatarsal	Multiple scooped-out lytic lesions with thin, overhanging, sclerotic margins that have coalesced resulting in substantial damage to the lateral aspect of the head and the lateral border of the lateral aspect of the distal articular surface.		
		Left 5^th^ metatarsal	Multiple scooped-out lytic lesions with thin, overhanging, sclerotic margins that have coalesced resulting in substantial damage to the lateral aspect of the head and the lateral border of the distal articular surface (Fig. 3a).		
		Right pedal sesamoid bone (x2)	Multiple scooped-out lytic lesions with sclerotic margins that have coalesced resulting in substantial damage to the elements (Fig. 3a).		
523	Young adult male	Right first distal pedal phalanx	Well-defined, scooped-out lytic lesion with sclerotic margins present on the dorsal aspect of the element, immediately adjacent to the proximal articular surface (Fig. 3d).	Only one joint is affected which is inconsistent with the distribution of rheumatoid arthritis. There are no proliferative changes, so it is unlikely to be erosive osteoarthritis. Nor is there involvement of the sacroiliac joints or spine, thus it is unlikely to be psoriatic arthritis or reactive arthritis.	Gout
535	Adult, sex unobservable	Right intermediate pedal phalanx	Scooped-out lytic lesions with overhanging, sclerotic margins that has resulted in the destruction of much of the lateral and planter aspect of the shaft. The lesion is located adjacent to the proximal joint surface (Fig. 3c).	The asymmetrical distribution of lesions in the feet is inconsistent with rheumatoid arthritis. There is no involvement of the sacroiliac joints or spine, thus unlikely to be psoriatic arthritis or reactive arthritis. There are no proliferative lesions associated with the erosive lesions, so it is unlikely to be erosive osteoarthritis.	Gout
		Right first metatarsal	Well-defined lytic lesions with sclerotic margins on the medial aspect of the head, located adjacent to the distal articular surface. There is also a ridge on located approximately 3 mm from the medial margin of the distal articular surface.		
		Left first metatarsal	There is a ridge located approximately 2 mm from the medial margin of the joint surface on the distal articular surface.		Hallux valgus
797	Middle adult female	Right first metatarsal	Well-defined, scooped-out lytic lesion with overhanging, sclerotic margins present on the medial aspect of the head, located immediately adjacent to the distal articular surface.	The asymmetrical distribution of the lesions is inconsistent with rheumatoid arthritis. There is no involvement of the sacroiliac joints or spine, thus unlikely to be psoriatic arthritis or reactive arthritis. There are no proliferative lesions associated with the erosive lesions, so it is unlikely to be erosive osteoarthritis.	Gout
		Right first distal pedal phalanx	Well-defined, scooped-out lytic lesion with overhanging margins, located on the medial aspect of the proximal articular surface. The lesion is primarily located on the joint margin, but the proximal articular surface is also affected.		

## 3 Results

### 3.1 Evidence for gout

**Fig. 2:**
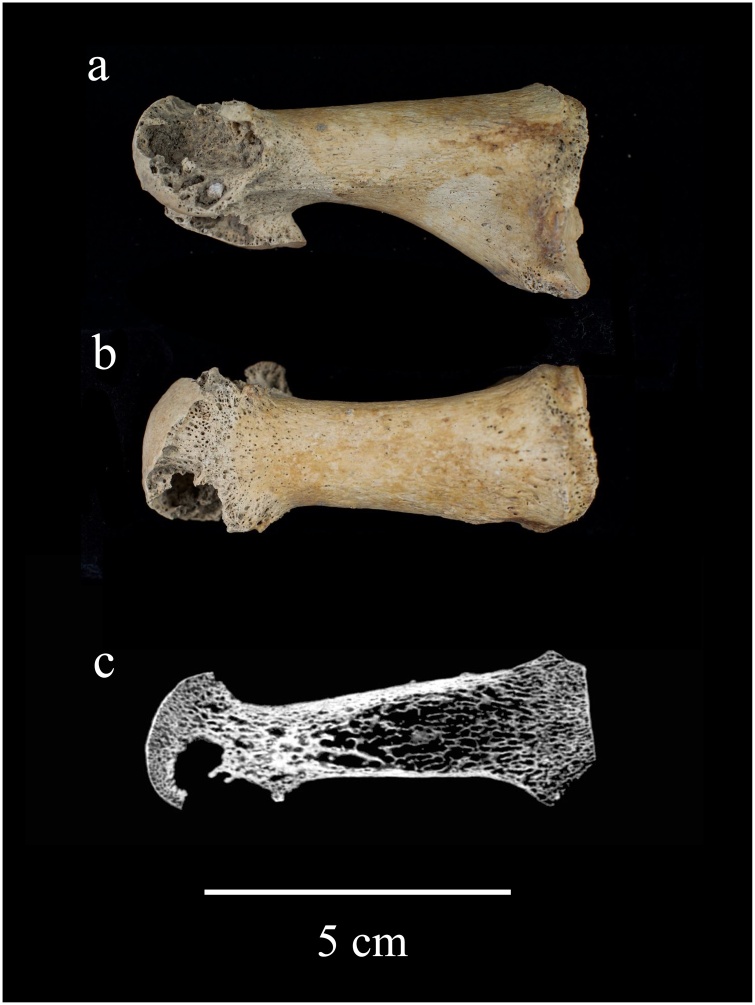
Lytic lesions caused by gout in an adult male individual from the Augustinian friary (PSN 522). Photograph of the a) medial and b) superior aspect of the right first metatarsal with c) μCT scan section through the axial plane. Photographs taken by Jenna Dittmar, μCT scan by Bram Mulder.

**Fig. 3:**
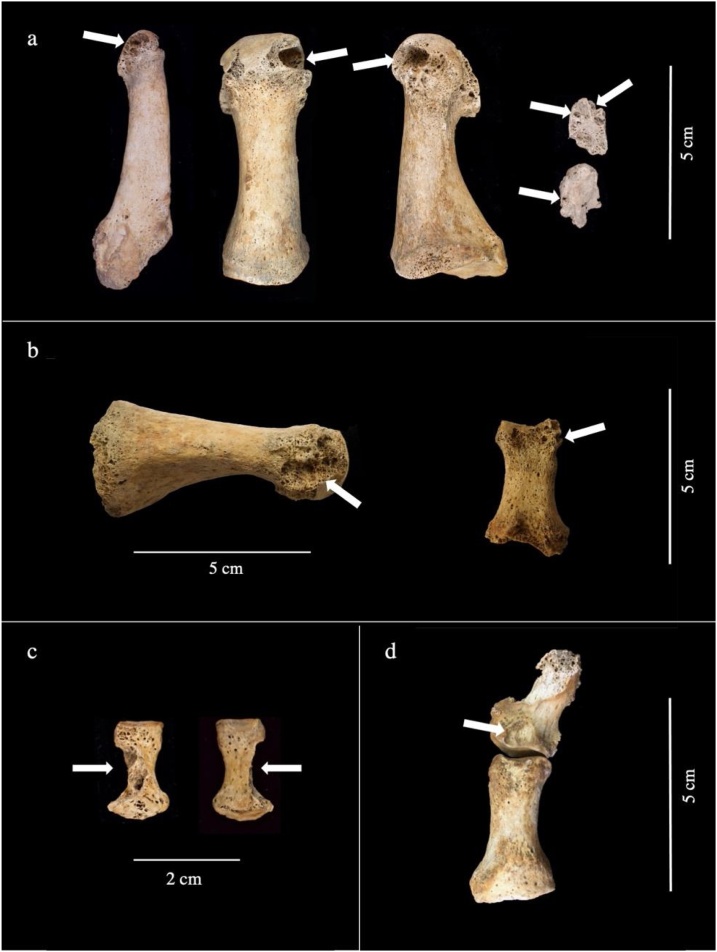
Well-defined, ‘scooped-out’ lytic lesions with sclerotic margins characteristic of gout (indicated by arrows) located on: a) the head and distal articular surface of the left fifth metatarsal (dorsal view), the left first metatarsal (dorsal and medial views) and on two pedal sesamoid bones (dorsal view) of an adult male individual from the Augustinian friary (PSN 522), b) the medial aspect of the head of the left first metatarsal (medial view) and on the distal aspect of the right first proximal pedal phalanx (plantar view) of an adult male individual from the Hospital (PSN 93), c) the lateral aspect of the shaft of a right intermediate pedal phalanx (plantar and dorsal view) of adult individual from the Augustinian friary (PSN 535), d) the dorsal aspect of the right distal pedal phalanx (dorsal view of the right proximal and distal pedal phalanx) of adult male individual from the Augustinian friary (PSN 523). Photographs taken by Jenna Dittmar.

Lytic lesions consistent with chronic tophaceous gout were identified in 3% (n = 6/177) of the individuals buried in one of the three burial grounds within Cambridge (see Fig. 2, Fig. 3 and Table 4). The highest prevalence rate was identified in the individuals buried the Augustinian friary (14 %, n = 3/21: friars, n = 2/11; wealthy lay people, n = 1/10), followed by those buried at the Hospital of St John with 3 % (n = 2/69) and All Saints by the Castle parish church with 2 % (n = 1/50). No skeletal evidence of gout was identified in those buried within the rural parish cemetery in Cherry Hinton. More males (n = 4) than females (n = 1) had lesions associated with gout (Table 4). A total of 123 individuals were able to be divided into distinct temporal categories: those that date to 11th-13th centuries (n = 52) and those to the 14th -15th centuries (n = 71). Five individuals with gout dated to the 14th or 15th century. It was not possible to accurately assign the other individual to a distinct time period. This suggests that gout may have been more prevalent in Cambridge during the 14th -15th centuries than in the 11th-13th centuries.

**Table 4:** Age, biological sex and date of death of individuals with gout.

PSN	Catalogue number	Site	Age	Sex	Date
42	2255	Hospital of St John	Middle Adult	Male	14th century
93	2276	Hospital of St John	Mature adult	Male	15th century
522	601	Augustinian friary (friar)	Old Adult	Male	14th century
523	539	Augustinian friary (friar)	Young adult	Male	15th century
535	351	Augustinian friary (laity)	Adult	Unobservable	14th century
797	EU 1.1.172	All Saints by the Castle Parish	Middle Adult	Female	10th–14th century

### 3.2 Gout and Hallux Valgus

**Fig. 4:**
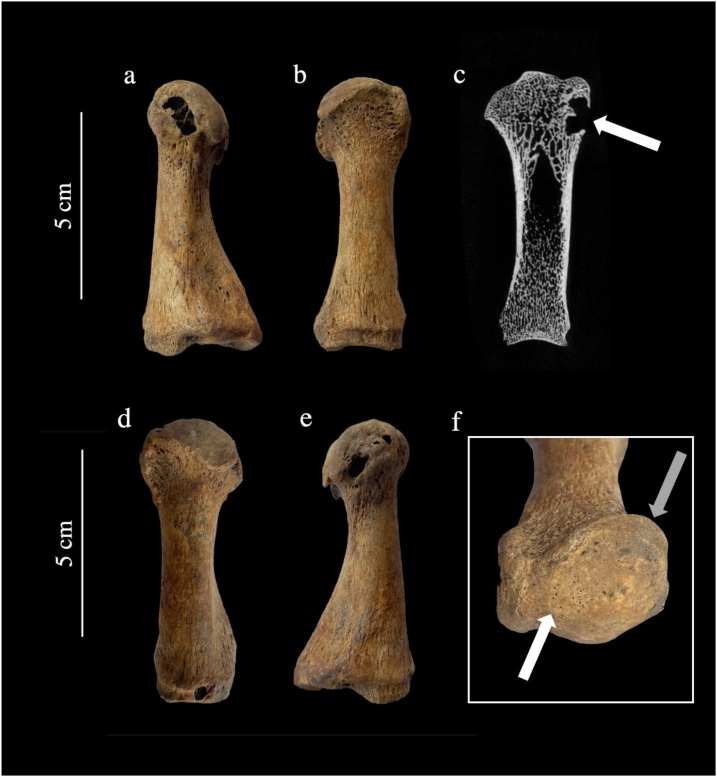
Right and left first metatarsals from an individual with hallux valgus and gout from the Hospital of St John the Evangelist (PSN 42). Photographs show the a) medial aspect of the left first metatarsal with a lytic lesion with thin, overhanging margins associated with gout, b) dorsal view of left first metatarsal showing lateral deviation of the distal joint surface as well as flattening of the medial aspect of the head, and anterolateral extension of the joint surface consistent with hallux valgus, c) micro-CT scan of the left first metatarsal showing the thin, overhanging, sclerotic margins of the lytic lesion that are consistent with gout (indicated by white arrow), d) dorsal view of right first metatarsal with lateral deviation of the distal joint surface consistent with hallux valgus, e) medial view of right first metatarsal with lytic lesions, f) distal articular surface of the left first metatarsal showing a ridge on the joint surface consistent with hallux valgus (indicated by white arrow) and an anterolateral extension of the joint surface (indicated by gray arrow). Photographs taken by Jenna Dittmar, μCT scan by Bram Mulder.

Two of the individuals with pathological changes consistent with gout also had hallux valgus. One individual (PSN 535) was buried in the Augustinian friary, and the other individual (PSN 42) was buried in the Hospital burial ground, giving an overall value of 1% (n = 2/177) (see Table 3 for a description or the observed lesions). Fig. 4 illustrates the skeletal lesions observed on PSN 42 that are consistent with each of these conditions.

## 4 Discussion

### 4.1 Gout in medieval England

The mean prevalence of chronic tophaceous gout in those buried in Cambridge was 3%. However, our ability to directly compare these results to the majority of other medieval sites is limited by the availability of true prevalence rates within the literature. Although it is not possible to directly compare true and crude prevalence rates, the overall trends observed in the Cambridge assemblages can be contextualized with what has been observed at other medieval sites. The prevalence of gout was highest in the Augustinian friary (14 %), followed by the Hospital (3 %), then All Saints parish cemetery (2 %), and absent in the rural parish of Cherry Hinton. When contextualized, the higher prevalence rate of gout in the Cambridge friary compared to the other sites within the town matches what we might expect. Cases of gout have frequently been observed in individuals that were buried in religious institutions, with crude prevalence rates as high as 14 % (Evans, 2014; WORD database, 2020a; Mays, 1991; Randerson et al., 2015; Swinson et al., 2010) (Table 5). The absence of gout in the rural parish burial ground of Cherry Hinton is also uncontroversial, as this finding is consistent with what has been observed at other rural parish burial grounds, such as Wharram Percy (Mays et al., 2007). Although not completely absent from rural areas (see Waldron, 2007), gout is more frequently recorded in urban areas, and in cemeteries that included high status individuals. As there has been little previous work assessing the epidemiology of gout anywhere in medieval Europe, further research is required in order to be able to fully contextualize and compare these findings.

**Table 5:** Frequency and prevalence rates of gout (including probable cases) in adult individuals from High/Late medieval skeletal assemblages in England.

Site	Location	Date (AD)	Sample size (adults only)	No. with gout	Crude prevalence rate (adults only)	Reference
**Hospitals**						
St Leonard, Newark Infirmary Hospital	Peterborough, Cambridgeshire	c. 1125−1538	123	1	1 %	Gilchrist and Sloane (2005)

**Monastic**						
Augustinian Priory of St Mary Merton †	London	1117–1538	643	3	0.5 %	WORD database (2020a)
Hull Augustinian Friary †	Kingston-upon Hull, Humberside	1316/7−1539	328 ‡	1	0.3 %	Evans (2014)
Augustinian Priory of St Leonard’s †	Torksey, Lincolnshire	Mid-14th c–1536	11	0	–	Booth (2002), Holst (2005a)
House of the Austin friars †	Leicester, Leicestershire	13th–16th c	15	0	–	Stirland (1981)
Blackfriars Friary †	Ipswich, Suffolk	1263−1538	226	1	0.4 %	Mays (1991)
Carmelite Priory	Northallerton, North Yorkshire	c. 1354−1538	7	1	14 %	Randerson et al. (2015)
Church of the Franciscans	Hartlepool, Cleveland	1240−1538	104	1	1 %	Daniels (1990)
Cistern abbey of St Mary Stratford Langthorne †	Stratford Langthorne, Essex	1135−1538/9	601 ‡	1	0.2 %	Barber et al. (2004)
Cistercian abbey of St Mary Graces †	London	1353−1538	283	0	–	WORD database (2020b)

**Rural Parish**						
St Martin’s Church	Wharram Percy, North Yorkshire	10th–16th c	360	0	–	Mays et al. (2007)
St Peter’s Church	Barton-on-Humber, Lincolnshire	11th–16th c	632 ‡ (periods C, C/D, D)	3	0.5 %	Waldron (2007)

**Urban Parish**						
St Mary Spital	London	c. 1120−1539	4120	2	<0.1 %	Connell et al. (2012)
Church of St Helen (Fishergate House)	York, North Yorkshire	11th–16th c	131	0	–	Holst (2005b)
St Helen-on-the-Walls	Aldwark, York, North Yorkshire	10th – mid -16th c	724	3	0.4 %	Dawes and Magilton (1980), Tucker (2015)
St Stephen’s Church	Fishergate, York, North Yorkshire	11th–mid 14th c	86	9	10.5 %	Tucker (2015)
St Gregory’s Priory, Northgate	Canterbury, Kent	1084−1537	69 ‡	1	1.5 %	Anderson (2001)

† These sites contain a mixture of lay and clerical individuals.

‡ Count includes juvenile individuals.

### 4.2 Social status, diet and gout in Cambridge

As has been cited in previous research, food consumption in medieval England can be indicative of social differentiation (see Driver, 2004; Rogers and Waldron, 2001; Woolgar, 2006). The consumption of alcohol and a protein-rich diet may partly explain the high prevalence rate of gout observed in the Augustinian friary and in the social elite who could afford to be buried in the friary. Although the presence of gout in the archaeological record is typically explained as a disease brought about by dietary excess, fasting can also be a trigger for a gout attack (Joosten et al., 2010). In accordance with the fasting days outlined in the ecclesiastical calendar, religious Orders often excluded meat from their diet, and routinely fasted for extended periods, although the sick were exempt from these rules (Andrews, 2006). This combination of rich diets with regular fasting likely contributed to the number of gouty attacks experienced by those residing in the friary, which in turn may have increased the high prevalence rate of skeletal lesions observed in this study.

The prevalence rates of gout for the individuals buried in the Hospital burial ground may have been bolstered by the presence of the university. The members of the largest and wealthiest colleges in Cambridge would consume a diet similar to that consumed by aristocratic households, with the staples of bread and ale along with large quantities of meat and fish (Lee, 2003). As the Hospital was the location to bury individuals that were not eligible to be buried in a parish burial ground, such as visiting scholars, it is possible that the individuals with evidence of gout may have been affiliated with the university. The Hospital is also the logical place for aged scholars to spend the final days of their lives as an inmate receiving charity, as a corrodian, an elderly layman that paid to live out the remainder of their life in the Hospital (Cessford, forthcoming a). The intense pain and physical impairment that can be caused by gout may have also been a contributing factor for why an individual would have been admitted to the Hospital.

Although it is difficult to reconstruct the exact diet consumed by the various individuals that made up the subgroups within the Hospital, textual sources suggest that the diets of the brethren and corrodians were similar and included bread made from the ‘finest wheat flour’, butter, eggs, fish and meat such as beef, veal, pork, piglets, mutton and chicken, plus occasionally goose and doves or pigeons (Cessford, forthcoming b). The diet consumed by the poor inmates in the Hospital is more difficult to discern, but it is likely that there would have been at least some overlap in diet between these subgroups.

Diet differed by social status and location during the High and Late medieval periods, and it is likely that the diets of those residing in many of the religious houses and colleges were different from the diets consumed by the average citizens. Access to foodstuffs would have varied in urban and rural environments. Meat is likely to have made up only a small part of the diet of most average citizens, although it became an increasingly important part of the diet across society from the late 14th century onwards (Woolgar et al., 2006: 100-1). Fish was also an important food item in the later medieval period, with improvements in fishing and trade allowing increasing access to marine fish products (Barrett et al., 2004). Fish was particularly important to those following religious observances, as it was an important protein source in the absence of meat (Serjeantson and Woolgar, 2006).

During the High and Late medieval periods, beer was routinely consumed by most members of society, regardless of social status. Alcohol, specifically beer and ale, have been shown to be associated with an increased risk of gout in modern population studies as both beer and ale contain yeast, which is high in purines (Choi, 2005). Historical sources suggest that up to one gallon per day per person was typically consumed (Woolgar et al., 2006: 11). The risk of hyperuricemia varies dependent on the kind and amount of alcohol consumed. However, research has shown that alcohol does not need to be present in these beverages to increase the risk of developing gout. Gibson et al. (1984) found that both alcoholic and non-alcoholic beer increase serum urate levels, which suggests that the ingestion of purine-rich drinks, even those without alcohol can increase uric acid levels. It is difficult to predict the effects of ale consumption on the medieval population, given that the purine content would have changed depending upon the amount of yeast removed prior to consumption (Hanawalt, 1986: 55)., It is likely, however, that the prolonged consumption of beer or ale, even those with low-alcohol content that were made and consumed during the High and Late medieval period, likely contributed to the development of gout in those from all social classes.

### 4.3 Co-occurrence of hallux valgus and gout

The co-occurrence of hallux valgus and gout was observed in two individuals, one lay person buried in the Augustinian friary and one individual buried at the Hospital. The presence of both these conditions in not uncommon in the clinical literature and concurrent research on these assemblages showed that at least 18 % of the population of medieval Cambridge had longstanding hallux valgus (Dittmar et al., forthcoming). Modern studies report that hallux valgus is significantly more common in patients with gout, with one study reporting hallux valgus was present in 41 % of individuals with gout (Roddy et al., 2008). Although clinical studies have not identified gout-specific or comorbid factors in the increased prevalence of hallux valgus in individuals that have gout (Roddy et al., 2015), it is not surprising that high status individuals from the High and Late medieval periods suffered from both conditions. Wealthy individuals that were able to afford fashionable footwear with pointed toe boxes that were popular in the Late medieval period were also more likely to be able to consume a rich diet that includes purine-rich foods. Based on the burial location, the adult individual (sex unobservable) buried in the friary is likely of higher social status. Typically, burial in the friary was limited to those who had ties to the friary, or to those who gave generous donations to the friary in exchange for burial.

The co-occurrence of these two conditions in an adult male buried in the Hospital burial ground is slightly more unusual. Radiocarbon dating indicates that this individual died between 1329−1430. This individual was buried in a standard inhumation burial in the Hospital cemetery, with no archaeological evidence to infer status. There are three scenarios that may account for why this individual was buried in the Hospital. One possibility is that this individual may have been an inmate who was physically impaired, possibly due to gout, who was in need of charitable care. Inmates were supposedly poor or infirm when admitted, however, that doesn't preclude individuals that were well-off and of high social status earlier in life, which may have contributed to the development of both of these conditions. It is also possible that this individual was a corrodian, a wealthy elderly individual that paid to spend the remainder of their life in a religious institution Cessford, forthcoming a). Textual sources mention that several corrodians were present at the Hospital (Rubin, 1987: 171–3; Underwood, 2008, xviii). Another possibility is that this man was a benefactor who requested to be buried in the Hospital following his death. While rich benefactors and patrons were likely to be buried in or near the Hospital Chapel, modest benefactors of the Hospital were probably buried in the detached cemetery (Cessford, forthcoming a).

### 4.4 Medieval medical texts in Cambridge that describe painful joints in the feet (Podagra)

The university presence in Cambridge has led to the preservation of medical texts from the medieval period. Analysis of these sources enables us to understand the views held by medical practitioners of the time on the cause and treatment of joint pain in the feet. In medieval medicine the Latin term for joint disease was *gutta artetica*, and the form affecting joints of the feet was *podagra*. There is evidence for teaching of medicine at the University in Cambridge after the Black Death of 1348−9, and Peterhouse College provided for two Fellows to study medicine. Two medical texts on the treatment of *podagra* can be linked to Peterhouse College.

In the manuscript Peterhouse MS 251 there is a treatise *De podagra,* written originally at the Benedictine Abbey of St Augustine’s, Canterbury, c.1100, but held at Peterhouse by the 15th century. The source material goes back to the *Therapeutica* of Alexander of Tralles, a Byzantine physician of the 6th century CE. *De podagra* describes joint pain as caused by an excess of humors flowing to the affected joint and discusses how to avoid it by a controlled diet, bloodletting in spring, bathing, and how to treat the swelling with topical medicines. Recipes are given for plasters, lotions, ointments and oils made of various combinations of herbs, spices, minerals, and animal parts. *De podagra* particularly recommends hermodactyl to be administered by mouth. This is the root of autumn crocus (*Colchicum autumnale*) and is used for gout into the modern era (as the medicine colchicine) (Field, 2015).

The second Peterhouse source for treating *podagra* was the *Rosa Anglica*, written by the 14th century English physician and surgeon John Gaddesden. This work was first printed in 1492, and there was a copy at Peterhouse. Gaddesden describes his own ointment for *podagra*. The main ingredient was dwarf elder (*ebulus*), cooked then mashed up with pork fat or may butter before application. He calls it *unguentum ebulinum* and states, ‘I tested it on a man with gout when nothing else worked’ (Gaddesden, 1492: sig.f8 verso).

Indeed, there is evidence that treatments for gout were available in Cambridge and applied at the Hospital, possibly even on some of the individuals whose skeletal remains we analyzed for this study. The accounts of the brother of the Hospital of St John, Master William Chandler, for 1505–09/10 include a list of herbs and plants for making *aqua vite* [‘water of life’, a spirit distilled from wine and herbs] (Underwood, 2008, xix; SJC archives D105.104). This was believed to help with many conditions, including gout [podagres, gowtes].

### 4.5 Suggestions for further research

Further research that assesses the epidemiology of gout in medieval Europe is needed in order to be able to fully contextualize and compare the findings presented here. However, the identification of gout in archeological assemblages remains challenging and further research that draws from modern clinical practice is needed to improve our ability to identify this condition in human skeletal remains. There are several conditions that can cause lytic lesions that are similar in appearance to those produced by chronic tophaceous gout (see Sections 2.2.1 and 2.2.2) and the location(s) (usually within the peripheral joints) of the lesions are relied upon to differentiate gout from other these conditions. Although a substantial proportion of individuals develop lesions in the first MTPJ, recent clinical research has shown that gout affects the axial skeleton, particularly the articular facets in the spine, much more commonly than previously thought (Jin et al., 2020; Lumezanu et al., 2012). The current criteria are also insufficient to identify less severe cases, or cases that are relatively early in the progression of the condition. Further clinically informed research that addresses these issues would be of benefit to the field of paleopathology.

## 5 Conclusion

Our investigation of gout in medieval Cambridge is one of the first to assess the epidemiology of this disease in pre-industrial Europe. The overall prevalence rate of 3% in Cambridge is higher than the reported 1 % prevalence for medieval Britain (Rogers, 2000; Waldron, 2007). However, as this is the first study to assess the true prevalence rate of gout for multiple cemeteries from the same town, our ability to directly compare these results to other medieval sites was limited by the lack of true prevalence rates reported within the literature.

Within the burial grounds in Cambridge, gout was most prevalent in those buried in the Augustinian friary, moderate in the other townsfolk, and absent in the rural parish cemetery. The difference in the prevalence rates amongst these subgroups can be partly explained by social differences in access to foodstuffs. The friars and the wealthy layfolk that paid to be buried in the Augustinian friary or the Hospital were more likely to consume alcohol and protein-rich diets than were the average citizens residing in and around the town. While we need to bear in mind the limitations of our sample size, gout appears to have been more prevalent in Cambridge during the 14th–15th centuries than it was during the 11th-13th centuries. This increase is likely due to a combination of factors that includes the establishment of the friary in the late 13th century, and an increase in affluence within the town as the university grew in size and prominence. Comparing burials from these different subpopulations within the town is a technique that others may find of potential if applied to future studies investigating gout at other medieval population centers.

Medical practitioners in the town were clearly aware of podagra, the medieval term most closely matching our modern understanding of gout. Texts held in the university at the time covered by our study mention options for how the disease might be treated, including the use of crocus roots that we now know contain colchicine. Records of the Hospital of St John even mention that they bought the ingredients for making *aqua vite*, which was believed to help against gout and other conditions.

## References

[bib0005] Anderson T., Hicks M., Hicks A. (2001). The human remains. St Gregory’s Priory, Northgate, Canterbury Excavations 1988-1991.

[bib0010] Andrews F. (2006). The Other Friars: The Carmelite, Augustinian, Sack and Pied Friars in the Middle Ages.

[bib0015] Barber B., Chew S., Dyson T., White B. (2004).

[bib0020] Barrett J., Locker A.M., Roberts C.M. (2004). ‘Dark Age Economics’ revisited: the English fish bone evidence AD 600-1600. Antiquity.

[bib0025] Booth W. (2002). The Human Remains.

[bib0030] Brooks S., Suchey J.M. (1990). Skeletal age determination based on the os pubis: a comparison of the Acsádi-Nemeskéri and Suchey-Brooks methods. Hum. Evol..

[bib0035] Buckberry J.L., Chamberlain A.T. (2002). Age estimation from the auricular surface of the ilium: a revised method. Am. J. Phys. Anthropol..

[bib0040] Buckley H.R. (2007). Possible gouty arthritis in Lapita-associated skeletons from Teouma, Efate Island, Central Vanuatu. Curr. Anthropol..

[bib0045] Buckley H.R. (2011). Epidemiology of gout: perspectives from the past. Curr. Rheum. Rev..

[bib0050] Buikstra J.E., Ubelaker D.H. (1994). Standards for Data Collection from Human Skeletal Remains. Arkansas archaeological survey research series No. 44.

[bib0055] Casson C., Casson M., Lee J.S., Phillips K. (2020). Compassionate Capitalism. Business and Community in Medieval England.

[bib0060] Cessford C. (2015). The St John’s Hospital cemetery and environs, Cambridge: contextualizing the medieval urban dead. Arch. J..

[bib0065] Cessford, C., 2017. Former Old Examination Hall, North Range Buildings, New Museums Site, Cambridge: an Archaeological Excavation. Cambridge Archaeological Unit Report 1377.

[bib0070] Cessford C., The sites, In press. Robb J., (ED), After the Plague: Health and History in Medieval Cambridge, forthcoming a, McDonald Institute monograph; Cambridge.

[bib0075] Cessford C., Historical setting, In press. Robb J., (ED), After the Plague: Health and History in Medieval Cambridge, forthcoming b, McDonald Institute monograph; Cambridge.

[bib0080] Cessford C., and Alexander C., The radiocarbon dating program and Bayesian modelling, In press. Robb J., Health and History in Medieval Cambridge, forthcoming, McDonald Institute Monograph; Cambridge.

[bib0085] Cessford C., Dickens A. (2005). Cambridge Castle Hill: excavations of saxon, medieval and post-medieval deposits, saxon execution site and a medieval coin hoard. Proc. Camb. Antiq. Soc..

[bib0090] Cessford C., Dickens A. (2005). The manor of Hintona: the origins and development of Church End, Cherry Hinton. Proc. Camb. Antiq. Soc..

[bib0095] Choi H.K. (2005). Diet, alcohol, and gout: how do we advise patients given recent developments?. Curr. Rheum. Rep..

[bib0100] Connell B., Gray Jones A., Redfern R., Walker D. (2012). A bioarchaeolgical study of medieval burials on the site of St Mary Spital: Excavations at Spitalfields Market, London E1, 1991–2007. MOLA monograph 60.

[bib0105] Craddock P.T., Gregory V.L. (1975). Excavation of the Graveyard of All Saints-AD-Castra.

[bib0110] Dalbeth N., Clark B., Gregory K., Gamble G., Sheehan G., Doyle A., McQueen F.M. (2009). Mechanism of bone erosion in gout: a quantitative analysis using plain radiography and computed tomography. Ann. Rheum. Dis..

[bib0115] Daniels R. (1990). The development of medieval hartlepool: excavations at church close, 1984–85. Arch. J..

[bib0120] Dawes J.D., Magilton J.R. (1980). The cemetery of St Helen-on-the-Walls. https://static1.squarespace.com/static/5c62d8bb809d8e27588adcc0/t/5cc85ebafa0d6046f3fc8df3/1556635342071/low+res+AY12-1-St+Helen+cemetery.pdf.

[bib0125] Dittmar, J.M., Mitchell, P.D., Inskip, S., Cessford, C., Inskip, S.A., Robb, J. Fancy shoes and painful feet: Hallux valgus and fracture risk in medieval Cambridge, England (under review at Int. J. Paleopath.). 10.1016/j.ijpp.2021.04.012 PMC8631459 34120868

[bib0130] Driver J.C., Jones O’Day S., Van Neer W., Revynck A. (2004). Food, status and formation processes: a case study from medieval England. Behaviour Behind Bones: the Zooarchaeology of Ritual, Religion, Status and Identity.

[bib0135] Dyer C. (2009). Making a Living in the Middle Ages: the People of Britain 850-1520.

[bib0140] Ellis D.M.B., Salzman L.F., Salzman L.F. (1948). Religious houses.

[bib0145] Evans D. (2014). Bring out your Dead! Burial rites and ritual in a medieval monastic cemetery. Lübeck und der Hanseraum: Beiträge zu Archäologie und Kulturgeschichte. Festschrift für Manfred Gläser.

[bib0150] Falys C.G., Prangle D. (2015). Estimating age of mature adults from the degeneration of the sternal end of the clavicle. Am. J. Phys. Anthropol..

[bib0155] Field V. (2015). The De Podagra (On Gout): a Pre-Gariopontean Treatise Excerpted from the Latin Translation of the Greek Therapeutica by Alexander of Tralles.

[bib0160] Fornaciari G., Giuffra V., Giusiani S., Fornaciari A., Villari N., Vitiello A. (2009). The ‘gout’ of the Medici, Grand Dukes of Florence: a palaeopathological study. Rheumatology.

[bib0165] Fornaciari G., Marinozzi S., Messineo D., Caldarini C., Zavaroni F., Iorio S., Sveva L., Capuani S., Catalano P., Gazzaniga V. (2019). A remarkable case of gout in the Imperial Rome: surgery and diseases in antiquity by osteoarchaeological, paleopathological, and historical perspectives. Int. J. Osteoarch..

[bib0170] Gaddesden, J., 1492. Rosa Anglica Practica Medicinae. Pavia.

[bib0175] Gentili A. (2003). Advanced imaging of gout. Semin. Musculoskel. Radiol..

[bib0180] Gentili A. (2006). The advanced imaging of gouty tophi. Curr. Rheum. Rep..

[bib0185] Gibson T., Rodgers A.V., Simmonds H.A., Toseland P. (1984). Beer drinking and its effect on uric acid. Rheumatology.

[bib0190] Gilchrist R., Sloane B. (2005). Requiem: the Medieval Monastic Cemetery in Britain.

[bib0195] Girish G., Glazebrook K.N., Jacobson J.A. (2013). Advanced imaging in gout. AJR: Am. J. Roent..

[bib0200] Hanawalt B.A. (1986). The Ties That Bound: Peasant Families in Medieval England.

[bib0205] Holst M. (2005). Osteological Analysis. http://www.yorkosteoarch.co.uk/pdf/0905.pdf.

[bib0210] Holst M. (2005). Fishergate House Artefacts and Environmental Evidence: The Human Bone. http://www.mgassocs.com/mono/001/rep_bone_hum1a.html.

[bib0215] Inskip S., Scheib C.L., Wohns A.W., Ge X., Kivisild T., Robb J. (2019). Evaluating macroscopic sex estimation methods using genetically sexed archaeological material: the medieval skeletal collection from St John’s Divinity School, Cambridge. Am. J. Phys. Anthropol..

[bib0220] İşcan M.Y., Loth S.R., Wright R.K. (1984). Age estimation from the rib by phase analysis: white males. J. For. Sci..

[bib0225] İşcan M.Y., Loth S.R., Wright R.K. (1985). Age estimation from the rib by phase analysis: white females. J. For. Sci..

[bib0230] Jin H.J., Son E.S., Kim D.H. (2020). The frequency of axial deposition in Korean patients with gout at a Tertiary Spine Center. Front. Med..

[bib0235] Joosten L.A.B., Netea M.G., Mylona E., Koenders M.I., Malireddi R.S., Oosting M., Stienstra R., van de Veerdonk F.L., Stalenhoef A.F., Giamarellos‐Bourboulis E.J., Kanneganti T.D. (2010). Engagement of fatty acids with toll‐like receptor 2 drives interleukin‐1β production via the ASC/caspase 1 pathway in monosodium urate monohydrate crystal–induced gouty arthritis. Arthr. Rheum..

[bib0240] Kim Y.S., Park E.H., Lee H.J., Koh Y.G. (2014). First metatarsophalangeal joint arthrodesis for the treatment of tophaceous gouty arthritis. Orthopaedics.

[bib0245] Kobayshi K., Deie M., Okuhara A., Adachi N., Yasumoto M., Ochi M. (2005). Tophaceous gout in the bipartite patella with intra-osseous and intra-articular lesions: a case report. J. Orthop. Surg..

[bib0250] Kuo C.F., Grainge M.J., Zhang W., Doherty M. (2015). Global epidemiology of gout: prevalence, incidence and risk factors. Nat. Rev. Rheum..

[bib0255] Lally M. (2008). 69 to 115 Church End, Cherry Hinton, Cambridgeshire: Post Excavation Assessment and Updated Project Design. Archaeological Solutions Report 3012.

[bib0260] Lee J.S. (2003). Feeding the colleges: Cambridge’s food and fuel supplies, 1450–1560. Econ. Hist. Rev..

[bib0265] Lumezanu E., Konatalapalli R., Weinstein A. (2012). Axial (spinal) gout. Curr. Rheumatol. Rep..

[bib0270] Martel W., Braunstein E.M., Borlaza G., Good A.E., Griffin P.E. (1979). Radiologic features of Reiter disease. Radiology.

[bib0275] Mays S. (1991). The Medieval Burials from Blackfriars Friary, School Street, Ipswich, Suffolk. English Heritage: Ancient Monuments Laboratory Unpublished Report, No. 16/91.

[bib0280] Mays S.A. (2005). Paleopathological study of hallux valgus. Am. J. Phys. Anthropol..

[bib0285] Mays S.A., Harding C., Heighway C. (2007). The Churchyard. Wharram: A Study of Settlement on the Yorkshire Wolds.

[bib0290] McDonald T., Doel P. (2000). Land at 69 to 115 Church End, Cherry Hinton, Cambs. Interim report Hertfordshire Archaeological Trust.

[bib0295] Miller E., Hatcher J. (2014). Medieval England: Rural Society and Economic Change 1086-1348.

[bib0300] Mitchell P.D., Brickley M. (2017). Updated Guidelines to the Standards for Recording Human Remains.

[bib0305] Neogi T., Jansen T.L.T.A., Dalbeth N., Fransen J., Schumacher H.R., Berendsen D., Brown M., Choi H., Edwards N.L., Janssens H.J.E.M., Lioté F., Naden R.P., Nuki G., Ogdie A., Perez-Ruiz F., Saag K., Singh J.A., Sundy J.S., Tausche A.-K., Vaquez-Mellado J., Yarrows S.A., Taylor W.J. (2015). 2015 gout classification criteria: an American College of Rheumatology/European League against Rheumatism collaborative initiative. Ann. Rheum. Dis..

[bib0310] Ortner D.J. (2003). Identification of Pathological Conditions in Human Skeletal Remains.

[bib0315] Phenice T.W. (1969). A newly developed visual method of sexing the os pubis. Am. J. Phys. Anthropol..

[bib0320] Randerson M.J., Watson J.E., Graham D.J., Caffell A., Cumberpatch C., Gidney L., Gutiérrez A., Jones J., Nolan J. (2015). Archaeological investigations at priory close, Northallerton, North Yorkshire. Yorkshire Arch. J..

[bib0325] Roddy E. (2011). Revisiting the pathogenesis of podagra: why does gout target the foot?. J. Foot Ankle Res..

[bib0330] Roddy E., Zhang W., Doherty M. (2008). Gout and nodal osteoarthritis: a case-control study. Rheumatology.

[bib0335] Roddy E., Muller S., Rome K., Chandratre P., Hider S.L., Richardson J., Blagojevic-Bucknall M., Mallen C.D. (2015). Foot problems in people with gout in primary care: baseline findings from a prospective cohort study. J. Foot Ankle Res..

[bib0340] Rogers J., Cox M., Mays S. (2000). The paleopathology of joint disease. Human Osteology in Archaeology and Forensic Science.

[bib0345] Rogers J., Waldron T. (1995). A Field Guide to Joint Disease in Archaeology.

[bib0350] Rogers J., Waldron T. (2001). DISH and the monastic way of life. Int. J. Osteoarch..

[bib0355] Rogers J., Waldron T., Dieppe P., Watt I. (1987). Arthropathies in palaeopathology: the basis of classification according to most probable cause. J. Archaeol. Sci..

[bib0360] Rome K., Survepalli D., Sanders A., Lobo M., McQueen F.M., McNair P., Dalbeth N. (2011). Functional and biomechanical characteristics of foot disease in chronic gout: a case-control study. Clin. Biomech..

[bib0365] Rothschild B.M., Heathcote G.M. (1995). Characterization of gout in a skeletal population sample: presumptive diagnosis in a micronesian population. Am. J. Phys. Anthropol..

[bib0370] Rubin M. (1987). Charity and Community in Medieval Cambridge.

[bib0375] Samsel M., Kacki S., Villotte S. (2014). Palaeopathological diagnosis of spondyloarthropathies: insights from the biomedical literature. Int. J. Paleopath..

[bib0380] Schwartz J.H. (2007). Skeleton Keys: An Introduction to Human Skeletal Morphology, Development, and Analysis.

[bib0385] Serjeantson D., Woolgar C.M., Woolgar C.M., Serjeantson, Waldon T. (2006). Fish consumption. Food in Medieval England.

[bib0390] Singh J.A., Strand V. (2008). Gout is associated with more comorbidities, poorer health-related quality of life and higher healthcare utilisation in US veterans. Ann. Rheum. Dis..

[bib0395] Stirland A., Mellor J.E., Pearce T. (1981). The human bone. CBA Research Report 35: The Austin friars, Leicester.

[bib0400] Sudoł-Szopińska I., Matuszewska G., Kwiatkowska B., Pracoń G. (2016). Diagnostic imaging of psoriatic arthritis. Part I: etiopathogenesis, classifications and radiographic features. J. Ultrason..

[bib0405] Swinson D., Snaith J., Buckberry M., Brickley M. (2010). High performance liquid chromatography (HPLC) in the investigation of gout in paleopathology. Int. J. Osteoarch..

[bib0410] Tucker K., McComish J.M. (2015). The human bone. Report Number AYW9: Roman, Anglian and Anglo-Scandinavian Activity and a Medieval Cemetery on Land at the Junction of Dixon Lane and George Street.

[bib0415] Underwood M.G. (2008). The Cartulary of the Hospital of St John the Evangelist, Cambridge.

[bib0420] Waldron T. (2007).

[bib0425] Waldron T., Buikstra J. (2019). Joint disease. Ortner’s Identification of Pathological Conditions in Human Skeletal Remains.

[bib0430] Woolgar C.M., Woolgar C.M., Serjeantson D., Waldon T. (2006). Meat and dairy products. Food in Medieval England.

[bib0435] Woolgar C.M., Serjeantson D., Waldron T. (2006). Food in Medieval England: Diet and Nutrition.

[bib0440] WORD database (2020). Museum of London. https://www.museumoflondon.org.uk/collections/other-collection-databases-and-libraries/centre-human-bioarchaeology/osteological-database/medieval-cemeteries/merton-priory.

[bib0445] WORD database (2020). Museum of London. https://www.museumoflondon.org.uk/collections/other-collection-databases-and-libraries/centre-human-bioarchaeology/osteological-database/medieval-cemeteries/st-mary-graces.

[bib0450] Zias J., Mitchell P.D. (1996). Psoriatic arthritis in a fifth century Judean Desert monastery. Am. J. Phys. Anthropol..

